# Genetic diversity of Spanish *Prunus domestica L*. germplasm reveals a complex genetic structure underlying

**DOI:** 10.1371/journal.pone.0195591

**Published:** 2018-04-09

**Authors:** Jorge Urrestarazu, Pilar Errea, Carlos Miranda, Luis G. Santesteban, Ana Pina

**Affiliations:** 1 Unidad de Hortofruticultura, Centro de Investigación y Tecnología Agroalimentaria de Aragón (CITA), Instituto Agroalimentario de Aragón-IA2 (CITA-Universidad de Zaragoza), Avda. Montañana, Zaragoza, Spain; 2 Departamento de Producción Agraria, Universidad Pública de Navarra, Campus Arrosadia s/n, Pamplona, Spain; Beijing Forestry University, CHINA

## Abstract

European plum (*Prunus domestica* L.) is an ancient domesticated species cultivated in temperate areas worldwide whose genetic structure has been scarcely analyzed to date. In this study, a broad representation of Spanish European plum germplasm collected in Northeastern Spain and a representative set of reference cultivars were compared using nuclear and chloroplast markers. The number of alleles per locus detected with the SSR markers ranged from 8 to 39, with an average of 23.4 alleles, and 8 haplotypes were identified. Bayesian model-based clustering, minimum spanning networks, and the analysis of molecular variance showed the existence of a hierarchical structure. At the first level, two genetic groups were found, one containing ‘Reine Claude’ type reference cultivars altogether with ca. 25% of local genotypes, and a second one much more diverse. This latter group split in two groups, one containing most (ca. 70%) local genotypes and some old Spanish and French reference cultivars, whereas the other included 24 reference cultivars and only six local genotypes. A third partition level allowed a significant finer delineation into five groups. As a whole, the genetic structure of European plum from Northeastern Spain was shown to be complex and conditioned by a geographical proximity factor. This study not only contributes to genetic conservation and breeding for this species at the national level, but also supports the relevance of undertaking similar tasks of collection and characterization in other unexplored areas. Moreover, this kind of research could lead to future coordinated actions for the examination of the whole European plum diversity, to define conservation strategies, and could be used to better understand the genetic control of traits of horticultural interest through association mapping.

## Introduction

The biological origin of European plum (*Prunus domestica* L.), a hexaploid species (2n = 6x = 48), is still controversial and remains uncertain [1, 2]. It is traditionally considered as an allopolyploid between *P*. *spinosa* (2n = 4x = 32) and *P*. *cerasifera* (2n = 2x = 16) [3–6], belonging to the *Prunus* genus within the *Prunoideae* subfamily of *Rosaceae* [7, 8]. This species probably originated at Western Asia, in the area south to the Caucasus Mountains and the Caspian Sea, which later travelled into Western Europe [8, 9]. Stone remnants indicate that *P*. *domestica* L. was used by humans, at least, 6,000 years ago, and it is known to have been widely cultivated in Roman times. This long history of domestication has resulted in a rich diversity of morphological characteristics and crop aptitudes [8].

Whereas the analysis of genetic diversity within and between populations in diploid species is nearly a routine task, the development of genetic studies focusing on polyploid species is hampered by several difficulties, mainly associated to the complexity of allelic scoring and molecular data analysis [10, 11]. Due to these limitations, *P*. *domestica* L. is one of the least studied species within the *Prunus* genus, and only few studies have focused on evaluating the genetic diversity and population structure of *P*. *domestica* L. germplasm [12–14]. However, learning the extent and structure of genetic variation in germplasm collections is a crucial step for the efficient conservation and utilization of biodiversity in cultivated crops [15–17]. Therefore, efforts should be made in order to better know the genetic variation and population structure in *P*. *domestica*, as already carried out for other fruit tree species, where SSR markers are widely applied in combination with clustering methods. Examples of such an approach can be found for apple [17–19], pear [16, 20], olive [21, 22], peach [23–24], apricot [25] or cherry [26] among many others.

Regarding the traditional use of European plum in Spain, the most detailed historical description can be found in “*Cartografía de Frutales en Hueso y Pepita*” [27], where valuable information on putative synonymies and geographical distribution of the main cultivars is provided at the regional level. Until the 1930s, the three main cultivars at the national level were ‘Reine Claude Verte’, ‘Reine Claude d’Oullins’ and ‘Reine Claude de Bavay’, the two latter being just two out of the wide number of derived-cultivars from ‘Reine Claude Verte’ [28–30]. Thereafter, local cultivars, ‘Arandana’, ‘Imperial Roja’ and ‘del País’, as well as other foreign cultivars such as ‘Reine Claude Violette’, ‘d’Ente’ or ‘Washington’ were reported as relevant in some specific regions at that time, but their cultivation at the national level was not widespread enough to be considered as ‘major cultivars’ [31, 32]. Despite the wide documentation for these cultivars in the country in the past, the present-day diversity existing in the country is widely unknown. This fact is especially true for mountainous areas of Northeastern Spain, which underwent a severe process of population decline from the second half of the last century, implying that most traditional farming areas were abandoned [33]. This situation encouraged the establishment of prospecting missions in mountainous areas from Northeastern Spain in order to recover this traditional material to be preserved in germplasm collections and to be further studied.

The specific aims of this study were to discern the extent of the genetic structure of the *P*. *domestica* L. from Northeastern Spain, as well as to investigate the genetic relationships between this local material and a diverse set of reference cultivars. In this study, we used for the first time codominant (SSR) and chloroplastic DNA (cpDNA) markers to elucidate the pattern of genetic diversity, structuration, and differentiation of *P*. *domestica* L. from the Northeast of the Iberian Peninsula.

## Materials and methods

### Plant material and DNA extraction

**Fig 1:**
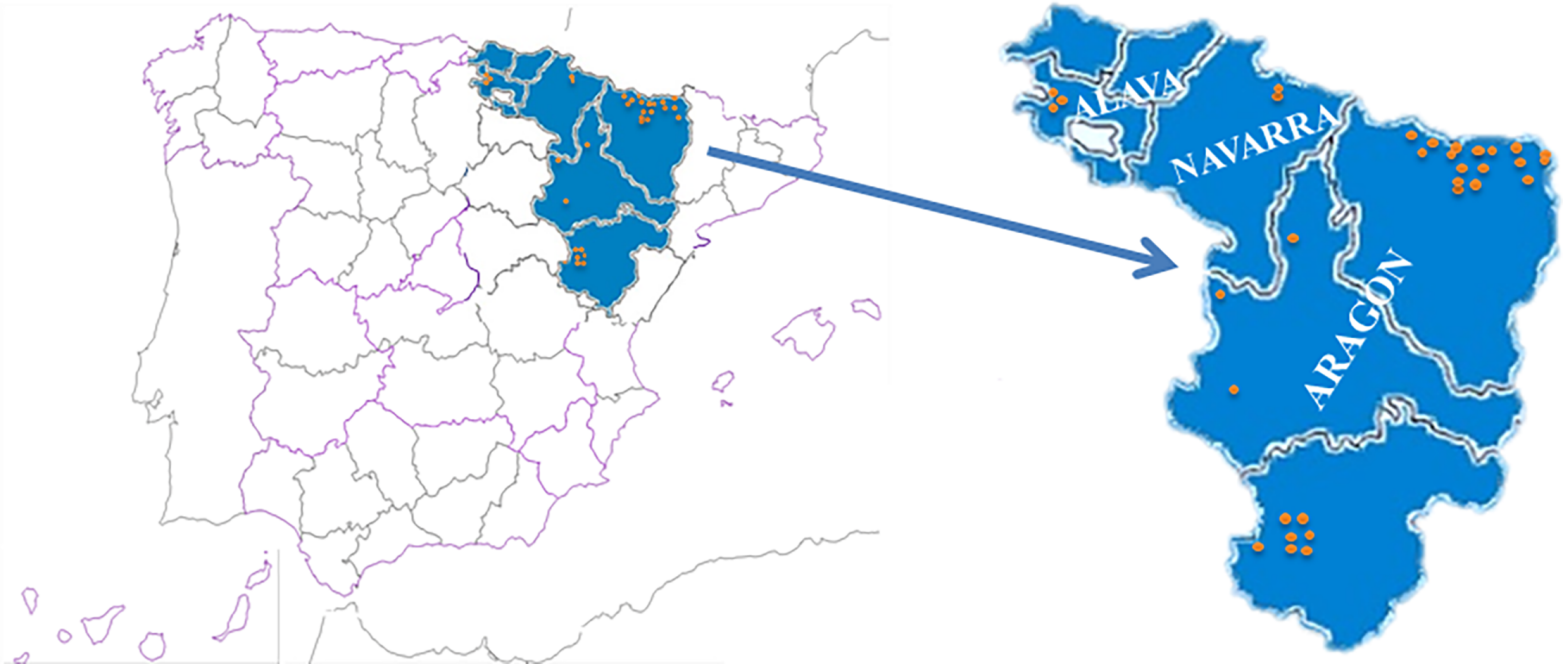
Geographic location of the collection sites of the local plum accessions included in this study. Collection localities prospected in areas from Northeastern Spain are indicated with orange dots.

This study considers 120 *P*. *domestica* accessions collected in areas from Northeastern Spain and an additional set of 46 cultivars used as a reference (Table 1). The prospection area includes a diversity of eco-geographical locations (Fig 1). All accessions, currently preserved at the CITA and Public University of Navarre collections, were prospected from singular trees that, at the moment of their collection, were actively cultivated (in backyards or small farms with the owner permission to conduct the study on this site) or were abandoned old trees for which specific permission was not required from three provinces of Aragon (Huesca, Zaragoza and Teruel), one from the Basque country (Alava) and Occidental Pyrenees in Navarra (S1 Table). The reference set was aimed at including: (i) old Spanish cultivars, (ii) old French cultivars (geographical proximity could have had important relationships with Spanish germplasm), (iii) a good representation of cultivars of the ‘Reine-Claude’-group, whose presence in the past was reported as very important in the country [28–30], and (iv) a diverse set of other international cultivars, some of them mentioned in literature as relatively important in the past in the country, at least at the regional-level [31].

**Table 1:** Listing of plum reference cultivars included in this study indicating group assignment by STRUCTURE analysis and their chloroplastic haplotypes.

Reference cultivar	Country of origin	*K* = 2	*K* = 3	*K* = 5	Haplotype
^1^Albatros	Hungary	G2.2	G3.3	G5.4	6
^1^California	USA	G2.2	G3.3	G5.4	1
^1^Kirke	UK	G2.1	G3.1	G5.1	1
^1^Lincoln	USA	G2.2	G3.3	G5.4	1
^1^Tragedy	USA	G2.2	G3.2	G5.2	1
^1^Tuleu gras	Romania	G2.2	G3.3	G5.4	6
^2^Amarouge	France	G2.2	G3.2	G5.3	1
^2^Campenca	France	G2.2	G3.2	G5.3	1
^2^De Montfort	France	G2.1	G3.1	G5.1	1
^2^Grosse Bleue	France	G2.2	G3.3	G5.4	1
^2^Impérial Epineuse	France	G2.1	G3.1	G5.1	5
^2^Marcarrière	France	G2.2	G3.3	G5.4	5
^2^Mariolet	France	G2.2	G3.2	G5.2	1
^2^Prune de Vars	UK	G2.1	G3.1	G5.1	1
^2^Royale Bleue	France	G2.2	G3.3	G5.2	7
^2^Saint Antonin	France	G2.2	G3.3	G5.3	1
^3^Anna Spath	Hungary	G2.2	G3.3	G5.4	1
^3^Arandana	Spain	G2.2	G3.3	G5.4	1
^3^De la Rosa	Spain	G2.2	G3.2	G5.3	1
^3^D'Ente	France	G2.2	G3.3	G5.4	1
^3^Pozegaka	USA	G2.2	G3.3	G5.4	1
^3^Ruth Gerstetter	Germany	G2.2	G3.3	G5.4	1
^4^Cacanska lepotica	Serbia	G2.2	G3.3	G5.4	5
^4^Cacanska rodna	Serbia	G2.2	G3.3	G5.4	5
^4^Ersinger	Germany	G2.2	G3.3	G5.4	1
^4^Jojo	Germany	G2.2	G3.3	G5.4	1
^4^Valor	Canada	G2.2	G3.3	G5.4	5
^5^Catalonia	Spain	G2.2	G3.2	G5.3	1
^5^Coe's Golden Drop	UK	G2.2	G3.3	G5.4	1
^5^Gran Prize	USA	G2.2	G3.3	G5.4	1
^5^Monsieur Hatif	France	G2.2	G3.2	G5.2	1
^5^Ontario	USA	G2.2	G3.3	G5.4	1
^5^Reine Claude Comte d'Altham	France	G2.1	G3.1	G5.1	1
^5^Reine Claude Diaphane	France	G2.1	G3.1	G5.1	1
^5^Reine Claude Rosée	France	G2.1	G3.1	G5.1	1
^5^Reine Claude Tardive de Chambourcy	France	G2.1	G3.1	G5.1	1
^5^Reine Claude Violette	France	G2.1	G3.1	G5.1	1
^5^Utility	UK	G2.2	G3.3	G5.2	1
^5^Washington	UK	G2.1	G3.1	G5.1	1
^6^President	UK	G2.2	G3.3	G5.4	1
^6^Reine Claude de Bavay	Belgium	G2.1	G3.1	G5.1	1
^6^Reine Claude d'Oullins	France	G2.1	G3.1	G5.1	1
^6^Reine Claude Verte	France	G2.1	G3.1	G5.1	1
^6^Stanley	USA	G2.2	G3.3	G5.4	5
^7^Questsche d'Italie	Italy	G2.1	G3.1	G5.1	1
^7^Victoria	UK	G2.2	G3.3	G5.4	1

Small numbers indicate the germplasm repositories where the reference cultivars were obtained

^1^ Research Institute for Fruit Growing and Ornamentals, Budapest Hungary

^2^French National Institute for Agricultural Research (INRA)

^3 ^Universidad Pública de Navarra (UPNA), Pamplona, Spain

^4^Institute of Pomology, Croatian Centre for Agriculture, Food and Rural Affairs, Zagreb, Croatia

^5^School of Agriculture, Policy and Development, University of Reading, UK

^6^Horofruticulture, CITA de Aragón, Zaragoza, Spain

^7^Pomology Department, CSIC, Zaragoza, Spain

Young leaves of each accession were collected and immediately conserved at -20°C until DNA extractions were performed. Total genomic DNA of each accession was isolated following the protocol described by Hormaza [34]. DNA concentration of each sample was quantified in a Nanodrop 1000 (Thermo Fischer Scientific, Wilmington, DE, USA), and working dilutions to 10 ng μl^-1^ final concentration were prepared.

### Genetic characterization

The genetic characterization of all the accessions was performed using both nuclear and chloroplast markers. With regards to nuclear markers, a set of 21 SSRs [35–39] was selected according to their location in the reference linkage map for *Prunus* T×E (almond ‘Texas’ × peach ‘Earlygold’). This SSR set contains three markers per chromosome, except for chromosomes 1, 2 and 4, for which two were included (S2 Table). The 21 SSR markers were amplified using four sets of multiplex PCR reactions, denoted as M01, M02, M03 and M04. Each multiplex was designed combining the molecular size (pb) of the fragments amplified for each SSR with different fluorescent dyes (S2 Table). The thermal profile for M01, M02, and M03 was performed as described in Dirlewanger et al. [38]. The four SSR markers included in M04 were amplified separately according to the thermal profiles proposed in their original references and combined after PCR. Fluorescently-labeled PCR products were separated using an ABI 3730 sequencer (Applied Biosystems, Foster City, CA, USA), and analyzed and sized with Peak Scanner Software version 1.0 (Applied Biosystems, Foster City, CA, USA).

Chloroplast analyses were based on the amplification of six non-coding cpDNA regions, and were performed using six consensus primer pairs [HK, K1K2, VL, CD, DT and CS [40, 41] and subsequently digested with three restriction enzymes: HinfI, TaqI and AluI (New England Biolabs). Three ng of total DNA were used for PCR amplifications, and 2 μl of PCR product were digested by 1.6 enzyme units in a mixture of 17 μl per sample. The reactions were incubated for 15 min at 65°C for TaqI, and 15 min at 37°C for HinfI and AluI, with a final inactivation for 20 min at 80°C. Restriction fragments were run on 2.5% agarose gels, stained with red dye, and visualized under UV light. Approximate restriction fragment size was estimated with a 100 bp ladder marker (Invitrogen).

### Diversity analysis

#### SSR diversity analysis

Since allele dosage determination in polyploid species is a complicated issue, we compared all multilocus genotypes scoring and recording the alleles as present/absent. Thus, for instance, AABBCC and AAAABC genotypes were both codified and included in the dataset as ABC. This way to codify the data is known as the ‘allelic phenotype’ approach [42, 43] and provides information on the presence of alleles, not on allele frequencies [44]. This approach has been shown to provide satisfactory results in recent population genetics works in polyploids [45–47]. The multilocus SSR profiles of all the accessions were compared pairwise in order to determine the genetic uniqueness of each accession and to quantify redundancy. The number of allelic phenotypes (A_P_), the number of observed alleles per locus (A_O_), the mean number of alleles per genotype (A_M_), the effective number of alleles (A_E_), and the number of rare alleles per locus (A_R_) were estimated for each SSR locus. In order to quantify the occurrence of rare alleles, two levels were considered, present in < 5% (A_<5%_) and < 1% (A_<1%_) of the genotypes.

A pairwise distance similarity matrix between all genotypes was calculated using Bruvo distance (D_B_) [48] through the ‘poppr’ R-package [49] on the R statistics platform [50], and graphically represented in minimum spanning networks (MSN) plots. Bruvo’s genetic distance takes distances between SSR alleles into account without the knowledge of allele copy number or the requirement that individuals be the same ploidy [51]. Bruvo’s distance ranges from 0, indicating identical genotypes, to 1, indicating maximum dissimilarity.

#### Chloroplast diversity analysis

As a complement to SSR diversity analysis, the polymorphism degree of the 18 possible cpDNA region/enzyme combinations was assessed. The study of chloroplast DNA variation using the PCR-RFLP has been shown to be useful and informative for studying chloroplast DNA diversity and phylogenetic relationships among *Prunus* species [12, 52–55]. For contingency reasons, a preliminary analysis of each primer-enzyme combination was performed in the set of the reference cultivars (46 genotypes). In a second analysis, only those combinations showing polymorphic fragments in the reference set were analyzed in the whole set of unique genotypes. The presence or absence of each restriction fragment at each polymorphic site was scored as binary data and used to identify chloroplastic haplotypes.

### Genetic structure inference

The software STRUCTURE version 2.3.4 [56] was used to estimate the number of hypothetical subpopulations (*K*) and to quantify the membership probability of each genotype to the inferred subpopulations. Analysis was performed under the admixture model and correlated allelic frequencies, and run using the recessive allele approach [57] codifying the genotypes following the recommendations provided in the software manual for polyploid species, successfully applied in previous studies [16, 19, 58–59]. The analysis was run for *K* values ranging from 1 to 10 inferred clusters, with 10 independent runs each, applying a burn-in period of 200,000 followed by 500,000 iterations. Structure Harvester ver. 0.6.93 [60], which implements the Δ*K* method defined by Evanno et al. [61], was used to estimate the most pertinent *K* value. Each genotype was assigned to the group for which it had the highest assignation probability (*qI*), considering a strong membership coefficient of a genotype to a particular group whenever *qI* ≥ 0.80 [16–19, 21], whereas when *qI* < 0.80 were considered as admixed genotypes. Placement of accessions on the inferred groups was determined using CLUMPP ver. 1.1 [62] and the CLUMPP output was directly used as input for Distruct ver. 1.1 [63] to graphically display the results.

### Genetic differentiation between and intra-group variability

The degree of differentiation between the genetic groups derived from STRUCTURE was estimated by performing analyses of molecular variance (AMOVA) using GenAlEx 6.5 [64, 65]. The statistical significance of the variance components was assessed using 1000 permutations. Moreover, pairwise PhiPT, an analog of Wright’s F_ST_ for dominant binary data [66], were calculated among groups.

The mean pairwise distance (MPD), i.e., mean of the pairwise PhiPT per genetic group, an index of the group differentiation relative to other genetic groups, was calculated for each group. The within-group sum of squares divided by the number of individuals in the group was applied as a normalized intra-group variability index (nSSWG) [66]. Additionally, the pairwise Bruvo genetic distances among all genotypes clustered within each group were represented by heat maps. Allelic intra-group variability measures were provided for each group defined by STRUCTURE, including the number of alleles (N_A_), the number of exclusive alleles (N_EA_) and the mean number of alleles per genotype (A_M_).

## Results and discussion

### SSR polymorphism and redundancy

The study of amplification products showed that eight out of the 21 SSR markers used were problematic in terms of absence of amplification product, insufficient fluorescence signal, low level of polymorphism or complex scoring pattern and, as a consequence, were excluded from subsequent analyses (S2 Table). Furthermore, two out of the remaining markers, BPPCT007 and UDP98-406, amplified two loci each. The amplification ranges (pb) for this pair of markers were 118–167 pb and 68–124 pb, respectively, and since it was difficult to delimit the allelic range for each locus, their use was exclusively restricted to the identification process, and excluded from further analyses. Therefore, despite the alleged high cross-transferability of SSR loci reported between *Prunus* species [38, 67–68], it may not be true when a larger set of samples is considered.

Based on the remaining 11 SSR markers, 135 unique genotypes were found within the whole set of accessions evaluated (120 local and 46 reference accessions), which corresponds to a 18% of duplication degree as a whole. Seventeen groups of SSR duplicates were identified. Two accessions had the same SSR profile than ‘Stanley’, whereas eight had the same profile than cultivars within the ‘Reine Claude’ group (i.e. ‘Reine Claude Verte’, ‘Reine Claude Tardive de Chambourcy’, ‘Reine Claude Diaphane’, ‘Reine Claude de Bavay’ and ‘Reine Claude d’Oullins’). The remaining groups of SSR duplicates were comprised exclusively by local accessions. It is worth noting that the latter SSR duplicates frequently included germplasm collected either in the Pyrenees or in the Iberian Cordillera, seldom including accessions collected from different areas. This fact may indicate that independent local selection processes could have been performed by the farmers from these areas in the past, seeking for adaptation to specific habitats across the heterogeneous Northeastern mountain landscape.

The presence of slight allelic differences was quantified by the pairwise Bruvo distance coefficient between genotypes, showing that 46 pairs of genotypes involving as a whole 24 differed in less than 0.05, which corresponded to a very few allelic mismatches. Slight allelic differences may result from potential genotyping errors, but also from putative somatic mutations, relatively frequent in long-lived tree species for which vegetative propagation has been used since ancient times [69–70].

### Genetic diversity

#### Assessment of genetic diversity using nuclear SSR markers

The 11 SSR markers proved to be highly diverse in terms of their degree of polymorphism, ranging from 8 (UDP96-008) to 39 (BPPCT025) alleles per locus, leading to a total number of 257 alleles (Table 2). The average number of alleles per locus (23.4) was slightly higher than those reported for Swedish (22.7; [14]) and Croatian germplasm (18.7; [13]), and lower than reported for French traditional germplasm (29) [12]. The mean number of alleles per genotype (A_M_) ranged from 2.74 (UDP98-409) to 4.89 (CPSCT026), with a mean value of 4.01. In spite of the high number of alleles found, the mean number of effective alleles (A_E_) was low (9.21), indicating that an important number of alleles appeared in the population at very low frequency. Thus, the proportion of rare alleles was high, with a mean percentage of 35.4% and 10.3% alleles per locus found respectively in less than 5% and 1% of the genotypes, and 32 alleles were unique (i.e. identified only in one genotype). This fact highlights the richness and allelic singularity that still can be found in traditional material, thus representing an important unexplored source of genetic variation.

**Table 2:** Allelic diversity of the European plum germplasm evaluated in this study.

SSR locus	LG	Motif	A_P_	A_O_	A_M_	A_E_	A_<1%_	A_<5%_
UDP96-005	1	(AC)_16_TG(CT)_2_CA(CT)_11_	112 (6.25)	35	4.19	13.82	2.86	40.00
CPPCT-029	1	(CT)_24_	36 (29.06)	17	3.34	6.11	0.00	41.18
UDP96-008	3	(CA)_23_	34 (34.09)	8	3.67	4.88	12.50	12.50
BPPCT-039	3	(GA)20	101 (9.02)	36	4.83	13.19	22.22	44.44
pchgms-2	4	(CT)_24_	72 (10.76)	19	3.59	6.87	10.53	42.11
CPSCT-005	4	(CT)_15_	90 (9.70)	25	3.89	8.38	20.00	52.00
UDP98-412	6	(AG)_28_	79 (16.66)	21	4.28	7.70	14.29	33.33
BPPCT-025	6	(GA)_29_	100 (8.80)	39	4.34	13.73	10.26	53.85
CPPCT-033	7	(CT)16	80 (14.91)	18	4,38	9.16	0.00	11.11
CPSCT-026	7	(CT)_16_	101 (12.59)	23	4.89	11.36	8.70	21.74
UDP98-409	8	(AG)_19_	62 (14.06)	16	2.74	6.17	12.50	37.50
*Mean*	–	–	78.82	23.36	4.01	9.21	10.35	35.43

A_P_, allelic phenotypes pointing out in brackets the frequency of the most common variant

A_O_, number of observed alleles

A_M_, mean number of alleles per genotype

A_E_, effective number of alleles

A_R_, number of alleles present in <1% and <5% of genotypes (as percentages of the total number of alleles for each SSR).

Genetic diversity was also quantified using the allelic phenotype approach, as this task is much more problematic in polyploids than in diploids, since more than two alleles per individual and locus are transmitted, potentially including multiple copies of a given allele [10]. In this study, the highest number of A_P_ corresponded, as expected, to those markers being the richest in detected alleles (Table 2). The most frequent A_P_ occurred in variable rates among markers, ranging between 6.25% (UDP96-005) and 34.09% (UDP96-008) of the genotypes.

#### Assessment of genetic diversity using chloroplast DNA (cpDNA) markers

Chloroplast DNA (cpDNA) variation was assessed using PCR-RFLP and, seven out of the 18 primer-restriction enzyme combinations resolved polymorphic fragments in the reference set of cultivars. When analyzed with those seven primer-restriction enzyme combinations, 13 polymorphic sites were detected (S3 Table), and the whole set of unique genotypes considered revealed eight different haplotypes (named from H1 to H8) (Table 3). This number of haplotypes is slightly higher than that found in 80 European plum cultivars from the French National collection, where five were detected using the same methodology [12]. In our study, H1 was by far the most prevalent haplotype, with a frequency of 0.86, whereas H5 was the second in frequency (0.08), and the remaining haplotypes included just two or a single genotype. Reference cultivars exhibited mainly haplotypes H1 and H5, the most frequent ones, although ‘Albatros’ and ‘Tuleu gras’ constituted H6 haplotype (Table 1).

**Table 3:** CpDNA haplotypes and frequencies in the *Prunus domestica* L. unique genotypes identified in this study.

Haplotypes	Haplotype combinations°	Unique genotypes	Frequency
H1	AAAAAAAAAAAAA	116	0.86
H2	AAAABCAAAAAAA	1	0.01
H3	BABABCAAABBBB	1	0.01
H4	BABABCAAACBBB	1	0.01
H5	BABABCCAACBBB	11	0.08
H6	BABBBDAAABBBB	2	0.01
H7	BABBBDBBBBBBB	2	0.01
H8	BBBBBDAAABBBB	1	001

° Each letter corresponds to a polymorphic restriction digestion site. Each unique combination of letters designates a haplotype.

A low degree of cpDNA variation can be due to historical events (i.e., number and type of the refugia, mode of recolonization of the species, etc.), but also to human-mediated activities [71]. The presence in our study of a prevalent haplotype is consistent with the results reported for *P*. *domestica* germplasm preserved at the French National Plum Collection [12]. These authors argued that the presence of one major haplotype may suggest a limited number of founders during the period that plum was first introduced into Western Europe.

### Genetic structure: Major divisions and substructuring of the diversity

#### Estimate of the number of hypothetical genetic groups

**Fig 2:**
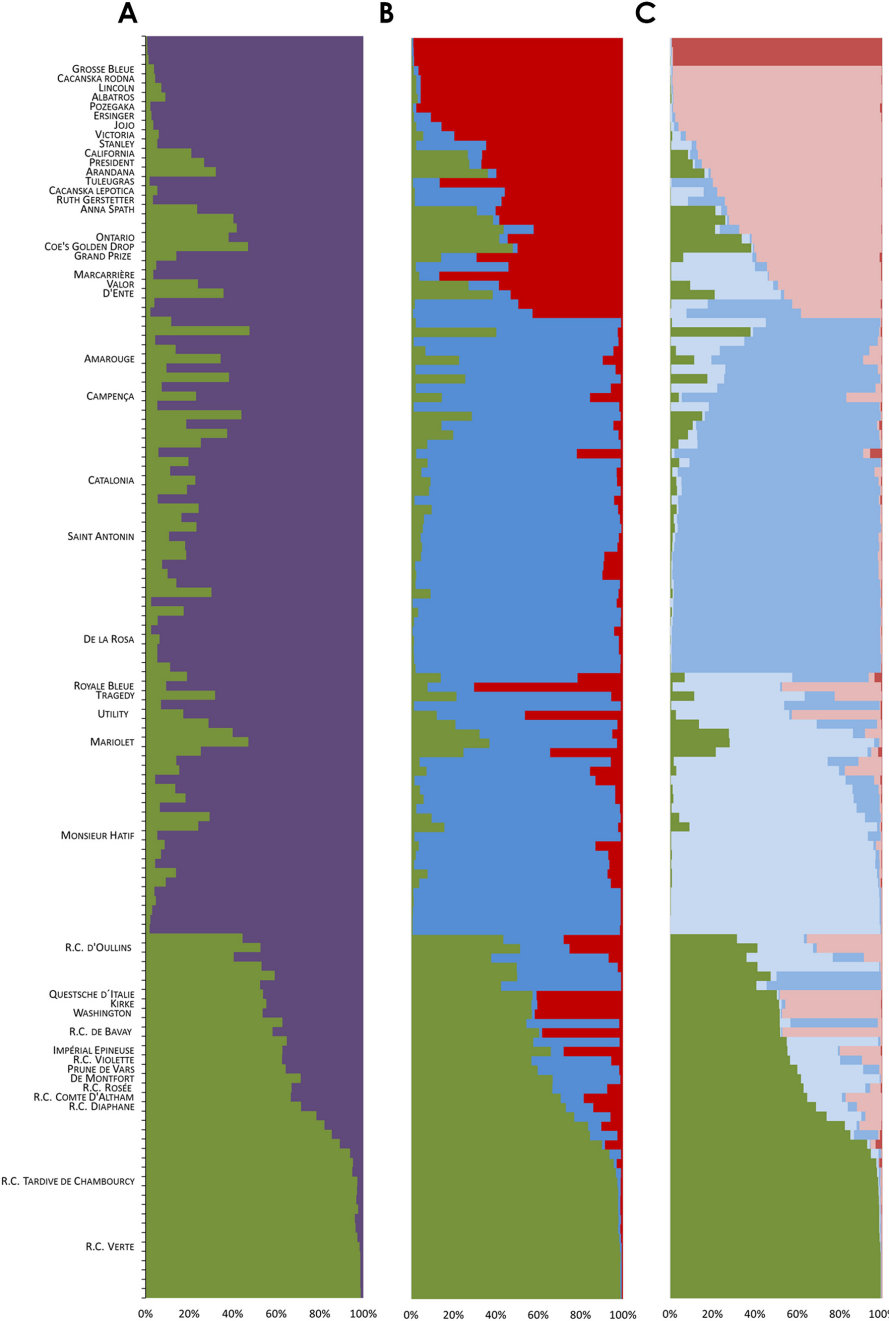
Graphical display of the results of the STRUCTURE analyses. Proportions of ancestry of 135 unique genotypes for groups inferred at *K* = 2 (A), *K* = 3 (B) and *K* = 5 (C). Each genotype is represented by a horizontal bar partitioned into two, three and five segments representing the estimated membership fraction in the groups obtained when *K* = 2, *K* = 3 and *K* = 5 were considered, respectively. Genotypes are presented in the same order in A, B and C. The groups inferred at *K* = 2 are depicted in green (G2.1) and in purple (G2.2). The groups inferred at *K* = 3 are depicted in green (G3.1), in blue (G3.2) and in red (G3.3). The groups inferred at *K* = 5 are depicted in green (G5.1), in light blue (G5.2), in dark blue (G5.3), in light pink (G5.4) and in dark pink (G5.5).

The analysis of the rate of change Δ*K* over the range of *K* values evaluated in STRUCTURE revealed that the germplasm could be divided into two and three groups, with a less pronounced peak found at *K* = 5 (Fig 2), Δ*K* values being 39.3, 33.1 and 12.4, respectively. Variation of the Δ*K* values as well as of the posterior log-probability of the data as a function of the number of *K* values is provided in S1 Fig. Group size for *K* = 2 was 37 (G2.1) and 98 (G2.2) genotypes (each group was named using first its *K* value followed by a cardinal number). For *K* = 3, group size was 67, 38 and 30 for G3.2, G3.1 and G3.3 respectively, and for *K =* 5 it varied from 3 (G5.5) to 41 (G5.3) (Table 4). The fact that all the *K* values resulted in asymmetric divisions may be indicative of the existence of a real population structure, and not resulting from a statistical artifact [56].

**Table 4:** Genetic diversity measures for each of the genetic groups defined with STRUCTURE at *K* = 2, *K* = 3 and *K* = 5. Number of genotypes (n), number of reference cultivars (n_R_), number of alleles (N_A_), number of exclusive alleles (N_EA_), mean pairwise distance (MPD), normalized intra-group variability index (nSSWG) and mean Bruvo distance between the genotypes clustered within each group.

Genetic group	n (*qI* > 0.80^a^)	n_R_ (*qI* > 0.80^a^)	N_A_	N_EA_	MPD	nSSWG	Mean D_B_
***K* = 2**							
G2.1	37 (19)	14 (2)	159	4	0.11	23.23	0.31±0.14
G2.2	98 (68)	32 (19)	253	98	0.11	36.44	0.54±0.11
***K* = 3**							
G3.1	38 (19)	14 (2)	159	6	0.14±0.01	23.90	0.39±0.14
G3.2	67 (50)	8 (4)	226	50	0.10±0.05	33.22	0.54±0.13
G3.3	30 (12)	24 (9)	193	12	0.10±0.05	38.71	0.50±0.08
***K* = 5**							
G5.1	34 (19)	14 (2)	149	3	0.23±0.14	22.53	0.30±0.15
G5.2	30 (17)	5 (1)	188	17	0.14±0.08	31.23	0.49±0.09
G5.3	41 (29)	5 (3)	184	16	0.13±0.08	31.84	0.49±0.08
G5.4	27 (12)	22 (12)	175	7	0.12±0.05	38.91	0.51±0.09
G5.5	3 (3)	0	39	7	0.27±0.12	13.78	0.58±0.08

^a^ Number of genotypes strongly assigned to the group (qI>0.80).

In species with a complex genetic background such as European plum, Bayesian clustering methods may detect genetic structures at different levels, which makes recommendable an examination of the results not only for the *K* value showing the highest Δ*K*, but also for some others showing relatively high values for this parameter in order to delineate further levels of substructure of the diversity. Therefore, the most likely divisions obtained for *K* = 2, *K* = 3 and *K* = 5 will be examined and compared throughout the subsequent sections. The consistency in the clustering of the genotypes between runs was examined to analyze the robustness of the divisions obtained at the three *K* values. The assignment of the genotypes was very consistent between runs, none shifting from genetic group when *K* = 2 and *K* = 3, and just 13 admixed genotypes showed slight discrepancy between runs when *K* = 5. These three partitioning levels were then compared in terms of their mean assignment probability of genotypes to the inferred groups for each of the three *K* values considered, as well as to the proportion of genotypes strongly assigned (*qI* ≥ 0.80) [17]. The mean probability of assignment for the genotypes to the inferred groups for the three *K* values was very high and almost identical (around 0.80). The proportion of genotypes strongly assigned differed between *K* values (Table 4), with 64%, 74% and 59% of the genotypes being strongly assigned when *K* values were 2, 3 and 5, respectively. The minimum spanning networks (MSN) based on Bruvo’s distance (S2 Fig) were consistent with the results obtained with the Bayesian clustering method at *K* = 2, *K* = 3 and *K* = 5, supporting the existence of the above mentioned genetic groups.

#### Placement of genotypes in the genetic groups

For *K* = 2, ca. 25% of the local genotypes clustered together in G2.1 with all the reference cultivars of the ‘Reine Claude’ group (Fig 2A), a varietal-group whose preponderance in the past at the national level in terms of geographical distribution and production has been broadly documented [28–31]. The remaining local genotypes (ca. 75%) clustered in G2.2 along with those reference varieties (32) not belonging to the ‘Reine Claude’ group (Table 4). This major structure remained greatly unchanged when the population was examined using *K* = 3 and *K* = 5, since the ‘Reine Claude’ group (G2.1) remained essentially unmodified (corresponding to G3.1 and G5.1), whereas higher *K* values allowed disentangling internal pattern of substructure of G2.2. The study of *Prunus domestica* L. germplasm maintained in the French National Plum Collection [12] also reported the structuring of ‘Reine Claude’ material as a separate group. Although ‘Reine Claude’ varietal-group was generally thought to have a genetic origin similar to other European plum morphological groups, Horvath et al. [12] argued that its genetic origin could be different, and our results support the hypothesis that ‘Reine Claude’ germplasm could have been originated from a distinct genetic background.

As mentioned above, when *K* = 3 was considered, G2.2 was partitioned into G3.2 and G3.3 (Fig 2B). The former group comprised ca. 70% of the local genotypes (Table 4), which appeared clustered in association with old Spanish and French reference cultivars such as ‘Monsieur Hatif’, ‘de la Rosa’, ‘Saint Antonin’ or ‘Catalonia’ [12, 31, 72–73]. By contrast, G3.3 included 24 reference cultivars, but only six local genotypes, all in *admixis* (Table 4; Fig 2B). Although G3.3 was very heterogeneous, it could be hypothesized that ‘Stanley’ (‘D’Ente’ × ‘Grand Duke’) could have played a significant role in the origin of this group, as some of the reference cultivars within G3.3 [e.g. ‘Cacanska lepotica’ (‘Stanley’ × ‘Rutt Gerstetter’), ‘Cacanska rodna’ (‘Stanley’ × ‘Rutt Gerstetter’), ‘Valor’ (‘Imperial Epineuse’ × ‘Grand Duke’), or ‘D’Ente’ (unknown parentage)] present differential degrees of relatedness to it. Moreover, the chloroplastic haplotypes for some of the reference cultivars of this group for which either their two parents or at least their maternal progenitors were analyzed share haplotype H5 (Table 1), the second most frequent out of the eight identified, which would reinforce the likeliness of this relatedness. Some examples of the latter are ‘Stanley’, ‘Cacanska lepotica’, ‘Cacanska rodna’ or ‘Valor’. Taking all the above into consideration, a certain level of genetic relatedness among some of the reference cultivars within this group may be hypothesized, though it cannot be confirmed, as their parentages are frequently unknown.

Last, when *K* = 5 was considered, G3.1 remained mainly unchanged, whereas G3.2 and G3.3 were split in two groups each, these subdivisions corresponding to, respectively, G5.2 and G5.3, and G5.4 and G5.5 (Fig 2C), suggesting that increasing *K* from 3 to 5 allowed discerning an additional substructure reflecting a finer delineation of this diversity. Groups G5.2, G5.3 and G5.4 were relatively similar in size (30, 41 and 27 genotypes, respectively), whereas G5.5 was a very small group, consisting of only three genotypes (Table 4). G5.2 and G5.3 are majorly comprised by local genotypes, as only 10 out of the 71 genotypes included are reference cultivars, mainly of French and Spanish origin. G5.4 includes most the genotypes clustered in G3.3, and G5.5 had just three genotypes, very strongly assigned. The latter is noteworthy as, despite the very small size of G5.5, two out of the three genotypes displayed two chloroplastic haplotypes occurring at a low frequency (H2 and H3).

All these results suggest that the structure of the plum local germplasm in Spain has been conditioned by a geographical proximity factor. Thus, ca. 95% of the local genotypes, clustered in three groups (G5.1, G5.2 and G5.3), where representatives of either the ‘Reine Claude’ group or old Spanish and French cultivars were found. The geographical proximity and historic connections between Spain and France, and the existence of ancient commercial roads and pilgrim’s routes since the Middle Ages could have favored the exchanges of cultivars between regions from both countries. Conversely, just a few local genotypes (ca. 5%) clustered together with other reference cultivars, which could result from the introduction in the late 1960s of cultivars such as ‘Stanley’, ‘President’, or ‘Ruth Gerstetter’ by commercial nurseries [31]. Therefore, to find only a residual presence of local genotypes related to such kind of germplasm is not surprising. By contrast, to find that ‘Arandana’, a very old and emblematic Spanish cultivar, clustered in the same group than ‘Stanley’, ‘President’, or ‘Ruth Gerstetter’ was unexpected. Therefore, the impact that ‘Arandana’ would have had in originating new local cultivars seems very limited, in spite of its past relevance in the country [31].

#### Distribution of the haplotypes into the groups inferred

The analysis of the frequency distribution of the chloroplastic haplotypes into the genetic groups defined by STRUCTURE revealed noticeable differences (S4 Table). When *K* = 2 was considered, G2.1 mostly comprised genotypes carrying haplotype H1 (ca. 95%), except for two admixed genotypes with haplotype H5, whereas G2.2 contained genotypes showing the eight haplotypes, including all those constituted just by one or two genotypes. With regards to the two most frequent haplotypes (H1 and H5), when *K* = 3 was considered, the frequency of occurrence of H1 was considerably lower in G3.3 (~67%) than those found for G3.1 and G3.2 (≥ 90%), whereas haplotype H5 was four times more frequent in G3.3 (20%) than in the other two groups (~5%). Similarly, the haplotype H5 was not equally distributed between groups when considering *K* = 5, being mainly concentrated in G5.2 and G5.4. Chi^2^ tests indicated significant differences in the distribution of the haplotypes between all pairs of groups except for the pairs G2.1-G2.2 (*P* = 0.120) and G3.1-G3.2 (*P* = 0.171).

Biparentally and maternally inherited markers such as, respectively, nuclear and cpDNA markers, have different inheritance modes and evolutionary rates. It is widely accepted that cpDNA markers reflect a past change in population variation, such as a population expansion or decline, whereas nuclear markers infer recent events in the population [74–75]. Therefore, it is not surprising than some haplotypes appear more frequently in some genetic groups inferred based on nuclear markers.

### Intragroup variability

The study of the intragroup variability revealed that the ‘Reine Claude’ group contained as a whole a lower level of diversity than other groups. This fact was supported by a much lower percentage of total alleles captured in comparison to those found in the other groups, and especially by a much lower presence of exclusive alleles harbored in this group. This trend was maintained irrespectively of the *K* value considered (Table 4). Moreover, the mean similarity of the genotypes from the ‘Reine Claude’ group ranged between 0.30 and 0.39 (depending on the *K* value considered), below the range observed for the other groups (between 0.49 and 0.58), and its intragroup variability index (nSSWG) was lower than the remaining ones, also mirroring its lower intragroup genetic diversity. Heat maps represented in S3 Fig provide a clear display of this trend, as the parts of the plot that include genotypes of the ‘Reine Claude’ group are much more homogeneous in color. All these results may indicate that local genotypes strongly clustered within this group are probably the result of conscious or unconscious hybridization processes between representatives of this genetic group.

The other group that presented distinct characteristics with this regard was G5.5, the genetic group integrated by just three genotypes. Despite its very small size, this group displayed seven exclusive alleles (Table 4) and belonged to two non-very common chloroplastic haplotypes (Table 3). It also presented the lowest nSSWG value, although this fact comes mainly from its extremely small size. These genotypes could therefore be hybrids between *P*. *domestica* L. and other *Prunus* species, since the occurrence of interspecific hybridizations, either natural or human-mediated, is relatively common within the genus *Prunus* [8, 12, 76]. Heat maps in S3 Fig also provide a clear graphical display of the distinct genetic nature of this group.

### Genetic differentiation between groups

Genetic differentiation between the groups obtained by the Bayesian model-based clustering method showed moderate differentiation (*P* < 0.001) for all the three *K* levels studied. Intergroup variation accounted for 11.4%, 10.8% and 12.7% of the total variation at *K* = 2, 3 and 5, respectively (Table 5). Regarding to MPD estimates, the groups exhibiting the highest differentiation relative to the other groups were G5.5 (0.27) and G5.1 (0.23) (Table 4). Pairwise PhiPT values at *K* = 3 resulted in moderate differentiation for the three pairs of genetic groups (0.10–0.14), whereas very high PhiPT estimates (up to 0.45) were found for some of the pairs of groups identified at *K* = 5 (Table 6). PhiPT values had been examined as they can help to identify unequal differentiation between some specific pairs that could remain hidden in the AMOVA and MPD estimates.

**Table 5:** Analyses of molecular variance (AMOVA) between the genetic groups defined with STRUCTURE at *K* = 2, *K* = 3 and *K* = 5.

Source of variation	df	Sum of square	Mean sum of square	Estimated variance	% of the variance	P(rand ≥ data)
***K* = 2**						
Among groups	1	263.90	263.90	4.29	11.41%	0.001
Within groups	133	4430.52	33.31	33.31	88.59%	—
Total	134	4694.42	—	37.60	100%	—
***K* = 3**						
Among groups	2	398.63	199.32	3.95	10.83%	0.001
Within groups	132	4295.79	32.54	32.54	89.17%	—
Total	134	4694.42	—	36.50	100%	—
***K* = 5**						
Among groups	4	594.29	148.57	4.60	12.72%	0.001
Within groups	130	4100.13	31.54	31.54	87.28%	—
Total	134	4694.42	—	36.14	100%	—

**Table 6:** Pairwise PhiPT values among the genetic groups defined with STRUCTURE at *K* = 2 (A), *K* = 3 (B) and *K* = 5 (C). Values below the diagonal line refer to the pairwise PhiPT values based on 1000 permutations and those above the diagonal line to the significance of PhiPT values.

**A**						
		**G2_1**	**G2_2**			
	**G2.1**	—	*0*.*001*			
	**G2.2**	0.114	—			
**B**						
		**G3_1**	**G3_2**	**G3_3**		
	**G3.1**	—	*0*.*001*	*0*.*001*		
	**G3.2**	0.136	—	*0*.*001*		
	**G3.3**	0.134	0.062	—		
**C**						
		**G5_1**	**G5_2**	**G5_3**	**G5_4**	**G5_5**
	**G5.1**	—	*0*.*001*	*0*.*002*	*0*.*001*	*0*.*002*
	**G5.2**	0.190	—	*0*.*001*	*0*.*001*	*0*.*001*
	**G5.3**	0.162	0.057	—	*0*.*001*	*0*.*001*
	**G5.4**	0.137	0.087	0.072	—	*0*.*009*
	**G5.5**	0.447	0.222	0.231	0.186	—

Analysing pairwise PhiPT in detail, it is worth highlighting that G5.2 and G5.3, the groups comprising ~70% of the local genotypes in close relationship mostly with old Spanish and French reference cultivars differentiated at 0.19 and 0.16 with the ‘Reine Claude’ group (G5.1), respectively. This high level of differentiation from ‘Reine Claude’ group was an unexpected finding, since it is the varietal-group reported to be the prevailing one in the past in terms of abundance and geographical distribution in Northeastern Spain. Taking that prevalence into account, it appeared sensible to expect a higher degree of relatedness and, consequently, a less differentiation between this group and those containing most of the Spanish traditional germplasm. The contribution of ‘Reine Claude’ group cultivars to create new variability appears to have been relatively limited to generating a relatively closed set of local cultivars, as they cluster with ~25% of local genotypes, but they apparently played a much lesser role in the origin of local genotypes in other groups. ‘Reine Claude’ varietal-group, having been crucial in European horticulture, with representatives as ‘Reine Claude Verte’ grown for more than five centuries [71, 77–78], has unquestionably played a decisive role shaping the genetic diversity of this species in Spain as well as at the European scale, but the intensity of this influence may differ depending on the geographical region and time of selection and on the aptitude sought.

Finally, G5.5 showed the highest MPD (0.27) (Table 4) and pairwise PhiPT values (ranging from 0.19 to 0.45) (Table 6). Although these estimates should be regarded with caution since could be biased due to the small size of G5.5, indicate a remarkable differentiation of these genotypes, and could be attributable to a putative hybrid status. This intergroup distinctness, altogether with the results described in the intragroup analysis, reinforces this hypothesis. However, this hybrid status should be confirmed using the same SSR markers in the characterization of a representative set of cultivars of different *Prunus* species, as well as of hybrids between *P*. *domestica* and other species.

## Concluding remarks

Our work demonstrates that, prospecting missions in unexplored areas may still be useful to recover an important source of diversity for this species, as local genotypes have been shown to enrich the genetic diversity held in varieties grown worldwide. Therefore, it would be advisable to perform similar tasks of collection and characterization, since understanding the extent and organization of diversity could promote efficient conservation actions, recovering ancient cultivars of potential interest, and ease their use into breeding programs in a near future. Nonetheless, the potential value of most local genotypes for the present-day market needs is largely unknown, since they have not been sufficiently characterized from an agronomic and consumer point of view. Hence, comprehensive phenotypic characterization based on standardized methods would be necessary in order to learn the commercial potentiality of these cultivars.

Our approach, based on Bayesian model-based clustering, minimum spanning networks, and the analysis of molecular variance, revealed the existence of a hierarchical structure in European plum germplasm from Northeastern Spain. At the first level, two genetic groups were found, one containing ‘Reine Claude’ type reference cultivars altogether with ca. 25% of local genotypes, but the study of genetic structure at further levels evidenced the existence of an additional internal substructure, that yielded up to five genetic groups. The inferred groups were clearly differentiated and showed noticeable differences in the allelic composition at the group level. Additionally, the fact that ca. 70% of the European plum local genotypes clustered with old Spanish and French reference cultivars indicates that population structure has been deeply conditioned by a geographical proximity factor, and underlines that the genetic background of an important part of the Spanish germplasm may differ from genepools of other origin. The genetic characterization reported herein not only constitutes the most comprehensive study of population structure from Spain, but also puts into value collection and characterization actions. Moreover, this kind of research could lead to future coordinated actions for the examination of the whole European plum diversity, to define conservation strategies, and could be used for defining an European core collection, useful to better understand the genetic control of traits of horticultural interest through association mapping.

## Supporting information

S1 Fig Exploration of *K* value for STRUCTURE analysis of the European plum germplasm evaluated in this study. (A) Estimates of the rate of change of the slope of the log likelihood curve (Ä*K*) calculated according to [61] are plotted against *K*. (B) Mean values of the log likelihood of the data given *K* is plotted against *K* for 10 simulations with a burn-in period of 200,000 followed by 500,000 iterations. The error bars show the standard deviations of the mean values.
(PNG)

S2 Fig **Minimum spanning networks (MSN) performed on Bruvo’s distances for all genotypes clustered in the groups defined by STRUCTURE at *K* = 2 (A), *K* = 3 (B) and *K* = 5 (C).** Each node represents one genotype. Edge thickness and color are proportional to genetic distance, while edge lengths are arbitrary.
(TIFF)

S3 Fig Heat maps based on Bruvo’s genetic distance matrix between all genotypes. Genetic groups inferred using STRUCTURE at *K* = 2 (A), *K* = 3 (B) and *K* = 5 (C). Color scales in the matrix indicate genetic distance between genotypes. Red indicates identical, while yellow indicates distantly related.
(TIF)

S1 Table Information of the unique genotypes used in this study. Collection information include name, collection site, specific longitude, latitude, approximate elevation, group placement by structure analysis with the mean qI values (when *K* = 2, *K* = 3 and *K* = 5 were considered) and the chloroplastic haplotypes.
(XLSX)

S2 Table Microsatellite code, linkage group, repeat type and PCR details of the 21 SSR markers analyzed in this study. (DOCX)

S3 Table Restriction patterns obtained with the primer pairs K1K2, HK, CD and VL and three restriction enzymes (AluI, HinfI and TaqI). Polymorphic sites are indicated in bold letters.
(DOCX)

S4 Table Frequency distribution of the chloroplastic haplotypes within the genetic groups defined by STRUCTURE at *K* = 2 (A), *K* = 3 (B) and *K* = 5 (C).
(DOCX)

## References

[1] Zohary D, Hopf M. Domestication of plants in the old world Oxford University Press, Oxford, UK; 2000.

[2] Reales A, Sargent DJ, Tobutt KR, Rivera D. Phylogenetics of Eurasian plums, *Prunus* L. section *Prunus* (*Rosaceae*), according to coding and non-coding chloroplast DNA sequences. Tree Genet Genomes. 2010; 6: 37–45.

[3] Crane MB, Lawrence WJC. The genetics of garden plants 4^th^ ed. Macmillan: London, UK, 1952 pp. 301.

[4] Watkins R. Cherry, plum, peach, apricot and almond In: Simmonds NW editor., Evolution of crop plants. Longman, London, UK, 1976 pp. 242–247.

[5] Watkins R. Plums, apricots, almonds, peaches, cherries genus *Prunus* In: Hora B, editor. The Oxford University encyclopedia of trees of the world. Oxford University Press, UK 1981 pp. 196–201.

[6] Faust M, Suranyi D. Origin and dissemination of plums. Hortic Rev. 1999; 23: 179–231.

[7] Potter D, Eriksson T, Evans RC, Oh S, Smedmark JEE, Morgan DR, et al Phylogeny and classification of *Rosaceae*. Plant Syst Evol. 2007; 266: 5–43.

[8] Topp BL, Russell DM, Neumüller M, Dalbó MA. Liu W. Plums In: Byme B, Badenes M, editors. Fruit breeding, handbook of plant breeding. Springer, New York; 2012 pp. 571–620.

[9] Cullinan FP. Improvement of stone fruits. In: Cullinan FP, Weinberger JH, editors. USDA yearbook of agriculture. Washington, DC; 1937. Pp. 605–702.

[10] Obbard DJ, Harris SA, Pannell JR. Simple allelic phenotype diversity and differentiation statistics for allopolyploids. Heredity. 2016; 97: 296–303. 10.1038/sj.hdy.6800862 16823402

[11] Dufresne F, Stift M, Vergilino R, Mables BK. Recent progress and challenges in population genetics of polyploid organisms: an overview of current state-of-the-art molecular and statistical tools. Mol Ecol. 2014; 23: 40–69. doi: 10.1111/mec.12581 24188632 10.1111/mec.12581

[12] Horvath A, Balsemin E, Barbot JC, Christmann H, Manzano G, Reynet P, et al Phenotypic variability and genetic structure in plum (*Prunus domestica* L.), cherry plum (*P*. *cerasifera* Ehrh.) and sloe (*P*. *spinosa* L.). Sci Hortic. 2011; 129: 283–293.

[13] Halapija Kazija D, Jelačić T, Vujević P, Milinović B, Čiček D, Biško A, et al Plum germplasm in Croatia and neighbouring countries assessed by microsatellites and DUS descriptors. Tree Genet Genomes. 2014; 10: 761–778.

[14] Sehic J, Nybom H, Hjeltnes SH, Gaši F. Genetic diversity and structure of Nordic plum germplasm preserved ex situ and on-farm. Sci Hortic. 2015; 190: 195–202.

[15] Lv J, Qi J, Shi Q, Shen D, Zhang S, Shao G, et al Genetic diversity and population structure of cucumber (*Cucumis sativus* L.). PLoS ONE. 2012; 7(10): e46919 doi: 10.1371/journal.pone.0046919 23071663 10.1371/journal.pone.0046919 PMC3470563

[16] Urrestarazu J, Royo JB, Santesteban LG, Miranda C. Evaluating the influence of the microsatellite marker set on the genetic structure inferred in *Pyrus communis* L. PLoS ONE. 2015; 10(9): e0138417 doi: 10.1371/journal.pone.0138417 26382618 10.1371/journal.pone.0138417 PMC4575082

[17] Urrestarazu J, Denancé C, Ravon E, Guyader A, Guisnel R, Feugey L, et al Analysis of the genetic diversity and structure across a wide range of germplasm reveals prominent gene flow in apple at the European level. BMC Plant Biol. 2016; 16(1):130 doi: 10.1186/s12870-016-0818-0 27277533 10.1186/s12870-016-0818-0 PMC4898379

[18] Pina A, Urrestarazu J, Errea P. Analysis of the genetic diversity of local apple cultivars from mountainous areas from Aragon (Northeastern Spain). Sci Hortic. 2014; 174: 1–9.

[19] Urrestarazu J, Miranda C, Santesteban LG, Royo JB. Genetic diversity and structure of local apple cultivars from Northeastern Spain assessed by microsatellite markers. Tree Genet Genomes. 2012; 8: 1163–1180.

[20] Miranda C, Urrestarazu J, Santesteban LG, Royo JB, Urbina V. Genetic diversity and structure in a collection of ancient Spanish pear cultivars assessed by microsatellite markers. J Am Soc Hortic Sci. 2010; 135: 428–437.

[21] Breton C, Pinatel C, Médail F, Bonhomme F, Bervillé A. Comparison between classical and bayesian methods to investigate the history of olive cultivars using SSR-polymorphisms. Plant Sci. 2008; 175: 524–32.

[22] El Bakkali A, Haouane H, Moukhli A, Costes E, van Damme P, Khadari B. Construction of core collections suitable for association mapping to optimize use of mediterranean olive (*Olea europaea* L.) genetic resources. PLoS ONE. 2013; 8(5): e61265 doi: 10.1371/journal.pone.0061265 23667437 10.1371/journal.pone.0061265 PMC3646834

[23] Aranzana MJ, Abbassi EK, Howard W, Arús P. Genetic variation, population structure and linkage disequilibrium in peach commercial varieties. BMC Genet. 2010;11: 69 doi: 10.1186/1471-2156-11-69 20646280 10.1186/1471-2156-11-69 PMC2915947

[24] Li XW, Meng XQ, Jia HJ, Yu ML, Ma RJ, Wang LR, et al Peach genetic resources: diversity, population structure and linkage disequilibrium. BMC Genet. 2013; 14: 84 doi: 10.1186/1471-2156-14-84 24041442 10.1186/1471-2156-14-84 PMC3848491

[25] Bourguiba H, Audergon JM, Krichen L, Trifi-Farah N, Mamouni A, Trabelsi S, et al Loss of genetic diversity as a signature of apricot domestication and diffusion into the Mediterranean Basin. BMC Plant Biol. 2012; 12: 49 doi: 10.1186/1471-2229-12-49 22510209 10.1186/1471-2229-12-49 PMC3511222

[26] Mariette S, Tavaud M, Arunyawat U, Capdeville G, Millan M, Salin F. Population structure and genetic bottleneck in sweet cherry estimated with SSRs and the gametophytic self-incompatibility locus. BMC Genet. 2010; 11:77 doi: 10.1186/1471-2156-11-77 20727153 10.1186/1471-2156-11-77 PMC2933703

[27] Herrero J. Cartografía de frutales de hueso y pepita Estación Experimental de Aula Dei, Zaragoza. Consejo Superior de Investigaciones Científicas–CSIC; 1964.

[28] Tabuenca MC, Iturrioz M. Description of European plum varieties III. Reina Claudia Verde. An Aula Dei. 1991a; 20: 153–163.

[29] Tabuenca MC, Iturrioz M. Description of European plum varieties IV. Reina Claudia de Oullins. An Aula Dei. 1991b; 20: 165–176.

[30] Tabuenca MC, Iturrioz M. Description of European plum varieties V. Reina Claudia de Bavay. An Aula Dei. 1991c; 20: 177–188.

[31] Herrero J, Iturioz M. Plum cultivars in Spain. An Aula Dei. 1971; 11: 165–199.

[32] Tabuenca MC, Iturrioz M. Description of European plum varieties I. Native varieties. An Aula Dei. 1991d; 20: 119–152.

[33] García-Ruiz JM, Lasanta T, Ruiz-Flaño P, Ortigosa L, White S, González C, et al Land-use changes and sustainable development in mountain areas: a case study in the Spanish Pyrenees. Landscape Ecol. 1996; 1: 267–277.

[34] Hormaza JI. Molecular characterization and similarity relationships among apricot (*Prunus armeniaca* L.) genotypes using simple sequence repeats. Theor Appl Genet. 2002; 104: 321–328. doi: 10.1007/s001220100684 12582704 10.1007/s001220100684

[35] Cipriani G, Lot G, Huang WG, Marrazzo MT, Peterlunger E, Testolin R. AC/GT and AG/CT microsatellite repeats in peach (*Prunus persica* (L.) *Batsch*): isolation, characterization and cross-species amplification in *Prunus*. Theor Appl Genet. 1999; 99: 65–72.

[36] Sosinski B, Gannavarapu M, Hager LD, Beck LE, King GJ, Ryder CD, et al Characterization of microsatellite markers in peach [*Prunus persica* (L.) Batsch]. Theor Appl Genet. 2000; 101: 421–428.

[37] Aranzana MJ, Pineda A, Cosson P, Dirlewanger E, Ascasibar J, Cipriani G, et al A set of simple-sequence repeat (SSR) markers covering the *Prunus* genome. Theor Appl Genet. 2002; 106: 321–328. 10.1007/s00122-002-1094-y 12647055

[38] Dirlewanger E, Cosson P, Tavaud M, Aranzana MJ, Poizat C, Zanetto A, et al Development of microsatellite markers in peach [*Prunus persica* (L.) Batsch] and their use in genetic diversity analysis in peach and sweet cherry. Theor Appl Genet. 2002; 105: 127–138. doi: 10.1007/s00122-002-0867-7 12582570 10.1007/s00122-002-0867-7

[39] Mnejja M, Garcia-Mas J, Howad W, Badenes ML, Arús P. Simple-sequence repeat (SSR) markers of Japanese plum (*Prunus salicina* Lindl.) are highly polymorphic and transferable to peach and almond. Mol Ecol Notes. 2004; 4: 163–166.

[40] Demesure B, Sodzi N, Petit RJ. A set of universal primers for amplification of polymorphic non-coding regions of mitochondrial and chloroplast DNA in plants. Mol Ecol. 1995; 4: 129–131. 7711952 10.1111/j.1365-294x.1995.tb00201.x

[41] Dumoulin-Lapègue S, Pemonge MH, Petit RJ. An enlarged set of consensus primers for the study of organelle DNA in plants. Mol Ecol. 1997; 6: 393–397. 9131816 10.1046/j.1365-294x.1997.00193.x

[42] Becher SA, Steinmetz K, Weising K, Boury S, Peltier D, Renou JP, et al Microsatellites for cultivar identification in *Pelargonium*. Theor Appl Genet. 2000; 101: 643–651.

[43] Esselink GD, Smulders MJM, Vosman B. Identification of cut rose (*Rosa hybrida*) and rootstock varieties using robust sequence tagged microsatellite site markers. Theor Appl Genet. 2003; 106: 277–286. doi: 10.1007/s00122-002-1122-y 12582853 10.1007/s00122-002-1122-y

[44] de Riek J, Everaert I, Esselink D, Calsyn E, Smulders MJM, Vosman B. Assignment tests for variety identification compared to genetic similarity-based methods using experimental datasets from different marker systems in sugar beet. Crop Sci. 2007; 47: 1964–1974.

[45] Sampson JF, Byrne M. Genetic diversity and multiple origins of polyploid *Atriplex nummularia* Lindl. (*Chenopodiaceae*). Biol J Linn Soc Lond. 2012; 105: 218–230.

[46] Vallejo-Marin M, Lye GC. Hybridisation and genetic diversity in introduced Mimulus (*Phrymaceae*). Heredity. 2013; 110:111–122. doi: 10.1038/hdy.2012.91 23169562 10.1038/hdy.2012.91 PMC3554457

[47] Rouger R, Jump AS. A seascape genetic analysis reveals strong biogeographical structuring driven by contrasting processes in the polyploid saltmarsh species *Puccinellia maritime* and *Triglochin maritime*. Mol Ecol. 2014; 23: 3158–3170. doi: 10.1111/mec.12802 24862943 10.1111/mec.12802

[48] Bruvo R, Michiels NK, D'Sousa TG, Schulenberg H. A simple method for calculation of microsatellite genotypes irrespective of ploidy level. Mol Ecol. 2004; 13: 2101–2106. doi: 10.1111/j.1365-294X.2004.02209.x 15189230 10.1111/j.1365-294X.2004.02209.x

[49] Kamvar Z.N, Tabima JF, Grünwald NJ. Poppr: an R package for genetic analysis of populations with clonal, partially clonal, and/or sexual reproduction. PeerJ. 2014; 2:e28. 10.7717/peerj.281 PMC3961149 24688859

[50] R Development Core Team. R: A Language and Environment for Statistical Computing. R Foundation for Statistical Computing, Vienna; 2016.

[51] Clark LV, Jasieniuk M. POLYSAT: an R package for polyploidy microsatellite analysis. Mol Ecol Resour. 2016; 11: 562–566. 10.1111/j.1755-0998.2011.02985.x 21481215

[52] Badenes ML, Parfitt DE. Phylogenetic relationships of cultivated *Prunus* species from an analysis of chloroplast DNA variation. Theor Appl Genet. 1995; 90: 1035–1041. doi: 10.1007/BF00222918 24173059 10.1007/BF00222918

[53] Mohanty A, Martin JP, Gonzalez LM, Aguinagalde I. Association between chloroplast DNA and mitochondrial DNA haplotypes in *Prunus spinosa* L. (*Rosaceae*) populations across Europe. Ann Bot. 2003; 92: 749–755. doi: 10.1093/aob/mcg198 14534199 10.1093/aob/mcg198 PMC4243615

[54] Panda S, Martin JP, Aguinagalde I, Mohanty A. Chloroplast DNA variation in cultivated and wild *Prunus avium* L.: a comparative study. Plant Breed. 2003; 122: 92–94.

[55] Bouhadida M, Martín JP, Eremin G, Pinochet J, Moreno MA, Gogorcena Y. Chloroplast DNA Diversity in *Prunus* and its implication on genetic relationships. J Am Soc Hortic Sci. 2007; 132: 670–679.

[56] Pritchard JK, Stephens M, Donnelly P. Inference of population structure using multilocus genotype data. Genetics. 2000; 155: 945–959. 10835412 10.1093/genetics/155.2.945 PMC1461096

[57] Falush D, Stephens M, Pritchard JK. Inference of population structure using multilocus genotype data: dominant markers and null alleles. Mol Ecol Notes. 2007; 7: 574–578. doi: 10.1111/j.1471-8286.2007.01758.x 18784791 10.1111/j.1471-8286.2007.01758.x PMC1974779

[58] Stöck M, Ustinova J, Lamatsch DK, Schartl M, Perrin N, Moritz C. A vertebrate reproductive system involving three ploidy levels: hybrid origin of triploids in a contact zone of diploid and tetraploid palearctic green toads (*Bufo viridis* subgroup). Evolution. 2010; 64: 944–959. doi: 10.1111/j.1558-5646.2009.00876.x 19863582 10.1111/j.1558-5646.2009.00876.x

[59] Lepais O, Muller SD, Ben Saad-Limam S, Benslama M, Rhazi L, Belouahem-Abed D, et al High Genetic diversity and distinctiveness of rear-edge climate relicts maintained by ancient tetraploidisation for *Alnus glutinosa*. PLoS ONE. 2013; 8(9): e75029 doi: 10.1371/journal.pone.0075029 24098677 10.1371/journal.pone.0075029 PMC3787099

[60] Earl DA, vonHoldt BM. Structure harvester: a website and program for visualizing Structure output and implementing the Evanno method. Conserv Genet Resour. 2012; 4:359–361.

[61] Evanno G, Regnaut S, Goudet J. Detecting the number of clusters of individuals using the software Structure: a simulation study. Mol Ecol. 2005; 14:2611–2620. doi: 10.1111/j.1365-294X.2005.02553.x 15969739 10.1111/j.1365-294X.2005.02553.x

[62] Jakobsson M, Rosenberg NA. CLUMPP: a cluster matching and permutation program for dealing with label switching and multimodality in analysis of population structure. Bioinformatics. 2007; 23:1801–1806. doi: 10.1093/bioinformatics/btm233 17485429 10.1093/bioinformatics/btm233

[63] Rosenberg NA. Distruct: a program for the graphical display of population structure. Mol Ecol Notes. 2004; 4:137–138.

[64] Peakall R, Smouse PE. Genalex6: genetic analysis in Excel. Population genetic software for teaching and research. Mol Ecol Notes. 2006; 6:288–295. 10.1093/bioinformatics/bts460 PMC3463245 22820204

[65] Peakall R, Smouse PE. GenAlEx 6.5: genetic analysis in Excel. Population genetic software for teaching and research-an update. Bioinformatics. 2012; 28: 2537–2539. doi: 10.1093/bioinformatics/bts460 22820204 10.1093/bioinformatics/bts460 PMC3463245

[66] Liorzou M, Pernet A, Li S, Chastellier A, Thouroude T, Michel G, et al Nineteenth century French rose (*Rosa* sp.) germplasm shows a shift over time from a European to an Asian genetic background. J Exp Bot. 2016; 67: 4711–4725. doi: 10.1093/jxb/erw269 27406785 10.1093/jxb/erw269 PMC4973750

[67] Wünsch A. Cross-transferable polymorphic SSR loci in *Prunus* species. Sci Hortic. 2009; 120: 348–352.

[68] Mnejja M, García_Mas J, Audergon JM, Arús P. *Prunus* microsatellite marker transferability across rosaceous crops. Tree Genet Genomes. 2010; 6: 689–700.

[69] Hartmann HT, Kester DE, Davies FT, Geneve RL. Plant propagation Principles and practices. 7th ed. Prentice-Hall: Upper Saddle River, New Jersey; 2002.

[70] Mudge K, Janick J, Scofield S, Goldschmidt EE. A history of grafting. Hortic Rev. 2009; 35:437–493.

[71] Palmé AE, Vendramin GG. Chloroplast DNA variation, postglacial recolonization and hybridization in hazel, Corylus avellana. Mol Ecol. 2002; 11(9):1769–1779. 12207726 10.1046/j.1365-294x.2002.01581.x

[72] Hedrich UP. The plums of New York. Ed. Albany J.B. Lyon Co., State printers, New York Agricultural Experiment Station; 1911.

[73] Leterme E, Lespinasse J. Les fruits retrouvés, patrimoine de demain: Histoire et diversité des espèces anciennes du Sud-Ouest Editions du Rouergue; 2008.

[74] Han Q, Higashi H, Mitsui Y, Setoguchi H. Distinct Phylogeographic Structures of Wild Radish (Raphanus sativus L. var. raphanistroides Makino) in Japan. PLoS ONE. 2015; 10(8): e0135132 doi: 10.1371/journal.pone.0135132 26247202 10.1371/journal.pone.0135132 PMC4527673

[75] Zhang X, Shen S, Wu F and Wang. Inferring genetic variation and demographic history of Michelia yunnanensis Franch. (Magnoliaceae) from chloroplast DNA sequences and microsatellite markers. Front Plant Sci. 2017; 8:583 doi: 10.3389/fpls.2017.00583 28484472 10.3389/fpls.2017.00583 PMC5399939

[76] Das B, Ahmed N, Singh P. *Prunus* diversity-early and present development: a review. Int J Biodivers Conserv. 2011; 3: 721–734.

[77] Lespinasse J, Leterme E. Growing fruit trees: novel concepts and practices for successful care and management New York: Norton WW and co; 2005.

[78] Gharbi O, Wünsch A, Rodrigo J. Characterization of accessions of ‘Reine Claude Verte’ plum using *Prunus* SSR and phenotypic traits. Sci Hortic. 2014; 169: 57–65.

