# Supplementary figures and images for: Genetic diversity of Spanish Prunus domestica L. germplasm reveals a complex genetic structure underlying
Source: PLoS One. 2018 Apr 9;13(4):e0195591. doi: 10.1371/journal.pone.0195591 (PMC5891032; doi:10.1371/journal.pone.0195591)

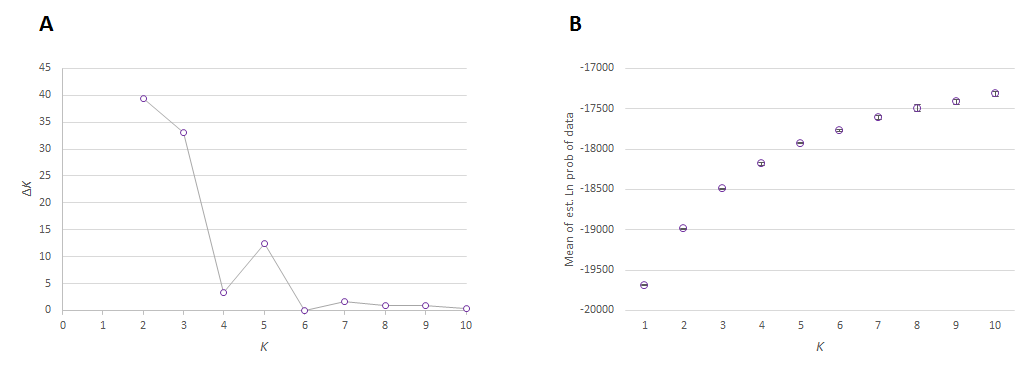

Supplement: S1 Fig — (A) Estimates of the rate of change of the slope of the log likelihood curve (ÄK) calculated according to [61] are plotted against K. (B) Mean values of the log likelihood of the data given K is plotted against K for 10 simulations with a burn-in period of 200,000 followed by 500,000 iterations. The error bars show the standard deviations of the mean values. (PNG) [file pone.0195591.s001.png]

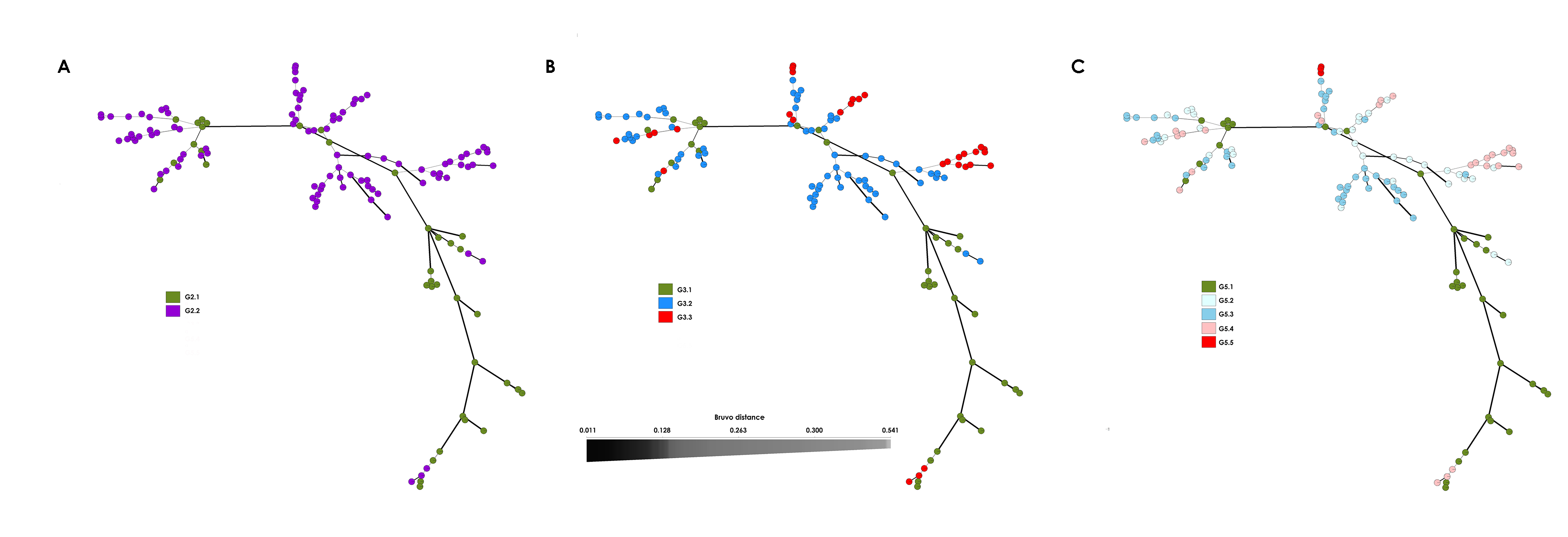

Supplement: S2 Fig — Minimum spanning networks (MSN) performed on Bruvo’s distances for all genotypes clustered in the groups defined by STRUCTURE at K = 2 (A), K = 3 (B) and K = 5 (C). Each node represents one genotype. Edge thickness and color are proportional to genetic distance, while edge lengths are arbitrary. (TIFF) [file pone.0195591.s002.tiff]
