# Supplementary material for: Genetic diversity of Spanish Prunus domestica L. germplasm reveals a complex genetic structure underlying
Source: PLoS One. 2018 Apr 9;13(4):e0195591. doi: 10.1371/journal.pone.0195591 (PMC5891032; doi:10.1371/journal.pone.0195591)
Supplement: S2 Table — (DOCX) [file pone.0195591.s005.docx]

**S2 Table**. Microsatellite code, linkage group, repeat type and PCR details of the 21 SSR markers analyzed in this study.

| **Locus** | **LG** | **Repeat type** | **Dye** | **Multiplex or Tª annealing** | **Reference** |
| --- | --- | --- | --- | --- | --- |
| UDP96-005 | 1 | (AC)_16_TG(CT)_2_CA(CT)_11_ | VIC | M03 | [35] |
| CPPCT-029 | 1 | (CT)_24_ | 6-FAM | M01 | [37] |
| *UDP97-402 | 2 | (AG)_17_ | PET | M01 | [35] |
| UDP98-406 | 2 | (AG)_15_ | VIC | M02 | [35] |
| BPPCT-007 | 3 | (AG)_22_(CG)_2_(AG)_4_ | 6-FAM | M03 | [38] |
| UDP96-008 | 3 | (CA)_23_ | PET | M02 | [35] |
| BPPCT-039 | 3 | (GA)_20_ | PET | M04 | [38] |
| pchgms-2 | 4 | (CT)_24_ | 6-FAM | M01 | [36] |
| CPSCT-005 | 4 | (CT)_15_ | NED | M04 | [39] |
| *BPPCT-017 | 5 | (GA)_28_ | PET | M03 | [38] |
| *BPPCT-037 | 5 | (GA)_25_ | VIC | 57 ºC | [38] |
| *BPPCT-038 | 5 | (GA)_25_ | NED | M03 | [38] |
| UDP98-412 | 6 | (AG)_28_ | NED | M02 | [35] |
| *UDP96-010 | 6 | (GT)21(GAGT)4(GA)18 | NED | 57 ºC | [35] |
| BPPCT-025 | 6 | (GA)_29_ | FAM | M04 | [38] |
| CPPCT-033 | 7 | (CT)_16_ | NED | M01 | [37] |
| CPSCT-026 | 7 | (CT)_16_ | VIC | M04 | [39] |
| *CPPCT-017 | 7 | (CT)_18_ | VIC | 60 ºC | [37] |
| UDP98-409 | 8 | (AG)_19_ | 6-FAM | M02 | [35] |
| *BPPCT-006 | 8 | (AG)_19_ | NED | 57 ºC | [38] |
| *CPSCT-018 | 8 | (CA)_5_(CT)_20_ | PET | 52 ºC | [39] |

* SSRs marked with an asterisk were excluded from subsequent analyses due to poor amplification. Polymorphic primers detecting more than one locus are underlined.
