# Supplementary material for: Genetic diversity of Spanish Prunus domestica L. germplasm reveals a complex genetic structure underlying
Source: PLoS One. 2018 Apr 9;13(4):e0195591. doi: 10.1371/journal.pone.0195591 (PMC5891032; doi:10.1371/journal.pone.0195591)
Supplement: S3 Table — Polymorphic sites are indicated in bold letters. (DOCX) [file pone.0195591.s006.docx]

**S3 Table.** Restriction patterns obtained with the primer pairs K1K2, HK, CD and VL and three restriction enzymes (AluI, HinfI and TaqI). Polymorphic sites are indicated in bold letters.

| **K1K2** | **Enzymatic restriction patterns in bp** | | | | | |
| --- | --- | --- | --- | --- | --- | --- |
| AluI-1 | 1453 | 400 | 292 | **259** |  |  |
| AluI-2 | 1453 | 400 | 292 | **276** |  |  |
| HinfI-1 | 585 | **388** | 376 | **289** |  |  |
| HinfI-2 | 585 | **400** | 376 | **289** |  |  |
| HinfI-3 | 585 | **400** | 376 | **322** |  |  |
| TaqI-1 | 788 | **287** | 273 | 217 |  |  |
| TaqI-2 | 788 | **385** | **300** | 273 | 217 |  |
| TaqI-3 | 788 | **765** | **307** | **300** | 273 | 217 |
| **HK** |  |  |  |  |  |  |
| AluI-1 | 600 | **386** | 223 |  |  |  |
| AluI-2 | 600 | **359** | 223 |  |  |  |
| AluI-3 | 600 | **339** | 223 |  |  |  |
| HinfI-1 | **769** | 600 | **229** | 171 |  |  |
| HinfI-2 | **690** | 600 | **234** | 171 |  |  |
| **CD** |  |  |  |  |  |  |
| TaqI-1 | 1247 | 933 | **613** | **353** | 200 | **160** |
| TaqI-2 | 1247 | 933 | **574** | **376** | 200 |  |
| TaqI-3 | 1247 | 933 | **815** | **376** | 200 |  |
| **VL** |  |  |  |  |  |  |
| AluI-1 | 700 | **558** | 368 |  |  |  |
| AluI-2 | 700 | **586** | 368 |  |  |  |
