# Supplementary material for: Genetic diversity of Spanish Prunus domestica L. germplasm reveals a complex genetic structure underlying
Source: PLoS One. 2018 Apr 9;13(4):e0195591. doi: 10.1371/journal.pone.0195591 (PMC5891032; doi:10.1371/journal.pone.0195591)
Supplement: S4 Table — Frequency distribution of the chloroplastic haplotypes within the genetic groups defined by STRUCTURE at K = 2 (A), K = 3 (B) and K = 5 (C). (DOCX) [file pone.0195591.s007.docx]

**S4 Table.** Frequency distribution of the chloroplastic haplotypes within the genetic groups defined by STRUCTURE at *K* = 2 (A), *K* = 3 (B) and *K* = 5 (C).

| **Groups** | **Chloroplastic haplotypes** | | | | | | | | |
| --- | --- | --- | --- | --- | --- | --- | --- | --- | --- |
|  | **H1** | **H2** | **H3** | **H4** | **H5** | **H6** | **H7** | **H8** |  |
| ***K* = 2** |  |  |  |  |  |  |  |  |  |
| G2.1 | 94.6 |  |  |  | 5.4 |  |  |  |  |
| G2.2 | 82.7 | 1.0 | 1.0 | 1.0 | 9.2 | 2.0 | 2.0 | 1.0 |  |
| ***K* = 3** |  |  |  |  |  |  |  |  |  |
| G3.1 | 94.7 |  |  |  | 5.3 |  |  |  |  |
| G3.2 | 89.6 |  |  | 1.5 | 4.5 |  | 3.0 | 1.5 |  |
| G3.3 | 66.7 | 3.3 | 3.3 |  | 20.0 | 6.7 |  |  |  |
| ***K* = 5** |  |  |  |  |  |  |  |  |  |
| G5.1 | 94.1 |  |  |  | 5.9 |  |  |  |  |
| G5.2 | 80.0 |  |  | 3.3 | 13.3 |  | 3.3 |  |  |
| G5.3 | 95.1 |  |  |  |  |  | 2.4 | 2.4 |  |
| G5.4 | 74.1 |  |  |  | 18.5 | 7.4 |  |  |  |
| G5.5 | 33.3 | 33.3 | 33.3 |  |  |  |  |  |  |
